# Polyaniline Nanoskein: Synthetic Method, Characterization, and Redox Sensing

**DOI:** 10.1186/s11671-020-03446-2

**Published:** 2020-11-13

**Authors:** Yoochan Hong, Hyun Soo Kim, Taeha Lee, Gyudo Lee, Ohwon Kwon

**Affiliations:** 1 Department of Medical Devices, Korea Institute of Machinery and Materials (KIMM), Daegu, 42994 Republic of Korea; 2 Department of Biotechnology and Bioinformatics, Korea University, Sejong, 30019 Republic of Korea

**Keywords:** Polyaniline, Nanoskein, Convertible nanoprobe, Glucose sensing, Redox states

## Abstract

Polyaniline nanoskein (PANS), which have polyaniline nanofibers, was developed. PANS was formulated via sequential extracting, heating, and swelling processes. The compositions of PANS have been analyzed using X-ray photoelectron spectroscopy, Fourier transform infrared spectroscopy, thermogravimetric analysis, and Brunauer–Emmett–Teller analysis, and the results of which indicate that PANS is composed of solely organic materials. Moreover, PANS has been shown convertible absorbance characteristics according to surrounding acidic environments, and using these characteristics, the possibility of PANS for sensing of surrounding redox state changes is presented.

## Introduction

Flower-shaped inorganic structures have been used for various applications such as catalysis [1, 2], biosensors [3, 4], and theragnostic agents [5]. There are some synthetic strategies for generating these complex structures. The synthesis using template is the first approach, for instance, liposome has been used as a soft template to guide the formation of dendritic Pt sheets [6, 7]. The second approach is based on the phenomenon of oriented attachment of primary nanoparticles when surfaces with similar atomic arrangements approach each other. The synthesis of dendritic PtRu nanoparticles forms faceted PtRu primary nanoparticles, which was based on this principle [8]. The third approach relies on the use of specific capping agents or surfactants to induce anisotropic growth of inorganic materials. The syntheses of Pt and Au multi-pods in the presence of poly(vinylpyrrolidone) or cetyltrimethylammonium bromide are such examples [9, 10]. In addition, after the first approach by Ge et al. [2], organic–inorganic hybrid flower-shaped structures have been intensively studied. In the process of synthesis for hybrid flower-shaped structures, the combinations of various proteins (e.g., enzymes [11] and DNA [12]) and metal ions (e.g., Cu [13], Ca [14, 15], and Mn [16]) have been used. In recent years, there were studies that synthesis of flower-shaped structures was based on polyaniline (PAni) using strong acid doping [17] and polyurethane nanofiber [18]. In addition, there were several reports on flower-shaped structures composed of organic materials, such as nitrogen-doped carbon materials [19, 20] and polyacrylonitrile [21].

In this study, we report a method for formulating organic flower-shaped structures, which is mostly composed of PAni, and its material properties. PAni, which is a representative conducting polymer, was used as an organic component in this study. PAni is well known for convertible optical properties due to its unique doping/dedoping or oxidation/reduction processes [22]. Using this phenomenon, our research group has reported that PAni-mediated nanostructures can be used as photo-thermal agents [23] and redox sensing probes [24, 25] for biomedical applications. In particular, the electronic band gap for PAni can be controlled by doping/dedoping states [26]. The difference between doping and dedoping states affects changes of optical spectra of PAni in the visible range [24]. These doping and dedoping states of PAni can be adjusted by various dopants, such as strong acids, Lewis acids, transition metals, and alkali ions [23].

**Fig. 1:**
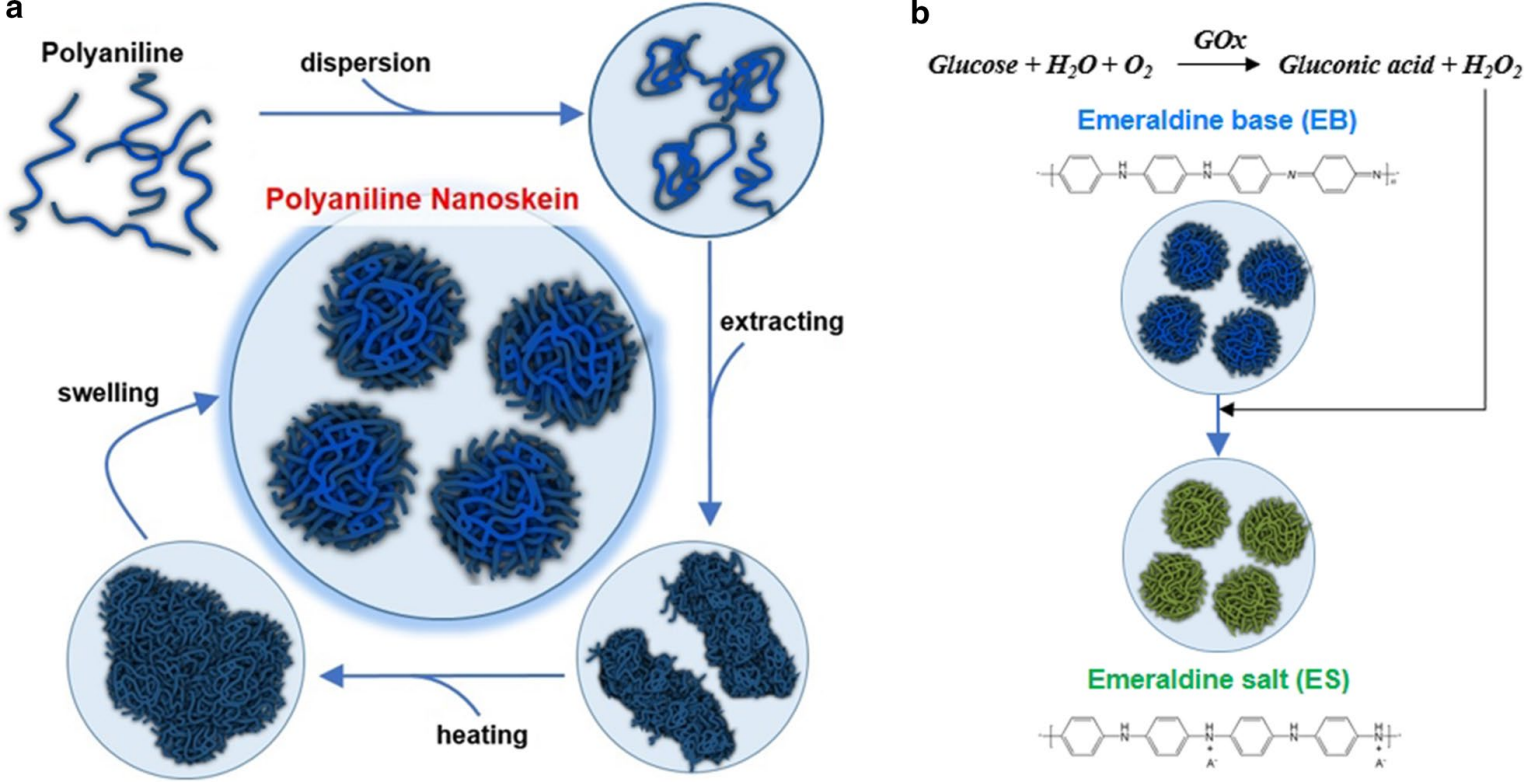
**a** The synthetic process of polyaniline nanoskein (PANS). **b** Glucose sensing process using PANS

We named flower-shaped PAni particles as PAni nanoskein (PANS) because the shape of PANS seems like a ball of yarn, which is composed of PAni nanofibers. Moreover, as-synthesized PAni appears to intrinsically form fibrillar and linear molecular structure such as thread. PANS was synthesized when we added benzyl ether (BE) to N-methyl-2-pyrrolidone (NMP) solution containing PAni, and this mixture was passed through heating and purification processes (Fig. 1a). And then, changes of surrounding redox states were measured based on color change of PANS, where optical properties were converted (Fig. 1b). However, many previously published reports have detected glucose using fluorescence ratiometric methods via various sensing mechanism [27–29]. To the best of our knowledge, up-to-date researches for detection glucose using convertible optical properties of PAni were deficient.

## Materials and Methods

### Materials

Polyaniline (PAni) Mw ~ 5000, benzyl ether (BE), glucose oxidase (GOx, from Aspergillus Niger, 145,200 units/g), and D-( +)-glucose were purchased from Sigma-Aldrich (St. Louis, MO, USA). N-Methyl-2-Pyrrolidone (NMP) and ethanol (EtOH) were purchased from Dae-jung, Korea. Dulbecco’s phosphate-buffered saline (DPBS) was purchased from Welgene, Korea, and buffer solution (pH 4) was purchased from Samchun, Korea. All other chemicals and reagents were of analytical grade. Ultrapure deionized water (DW) was used for all the synthetic processes.

### Synthesis of Polyaniline Nanoskein (PANS)

The synthetic process of immaculate product was as follows: PAni (250 mg) was dissolved in NMP (20 mL), and then, BE (40 mL) was added. The mixture was preheated to 200 °C for 1 h and heated to 300 °C for 30 min. The reactant was cooled in room temperature for 3 h. The resultant solution was washed with EtOH and separated the precipitant by centrifugation at 3000 rpm for 10 min. Then, the solution was re-dispersed in EtOH. Next, the solution dissolved in EtOH was dialyzed using dialysis membrane (MWCO: 3,500 Spectra/Por® 6, SPECTRUM® LABORATORIES, Rancho Dominguez, CA, USA) for 24 h. After dialysis, the solution was centrifuged at 15,000 rpm for 30 min. The precipitant was re-dissolved in 30 mL of DW. The final concentration of PANS was calculated as 8.33 mg/mL.

### Characterization of PANS

The morphology of PANS was evaluated with transmission electron microscopic (TEM, JEM-1011, JEOL Ltd, Japan) and scanning electron microscopic (SEM, JSM-6701F, JEOL Ltd, Japan) imaging. X-ray photoelectron spectra were recorded using a K-alpha system (Thermo Fisher Scientific, Waltham, MA, USA). Fourier transform infrared spectra (FT-IR Spectrum Two, PerkinElmer, Waltham, MA, USA) analysis was performed to confirm the characteristic bands of the synthesized PANS, and the size distribution of the PANS was analyzed by a dynamic light scattering (ELS-Z, Otsuka electronics, Japan) method. Furthermore, the surface area and pore volume of PANS were measured by a Brunauer–Emmett–Teller analyzer (Autosorb-iQ 2ST/MP, Quantachrome Instruments, Boyton Beach, FL, USA), and the weight quantity of PANS was analyzed with a thermogravimetric analyzer (SDT-Q600, TA instrument, New Castle, DE, USA). Moreover, the absorbance of PANS was analyzed using a UV–Vis spectrophotometer (Optizen 2120UV, MECASYS Co., Korea).

### Glucose Sensing Using PANS

First of all, the concentration of GOx was adjusted into 15 mg/mL using DPBS, and glucose was diluted with pH 4 buffer solution. The resultant concentrations of glucose were set at 40, 20, 10, 5, and 1 mg/mL, respectively. And then, 1 mL of PANS (1.67 mg/mL) was mixed with 20 μL of GOx (15 mg/mL) and 80 μL of glucose (40, 20, 10, 5, and 1 mg/mL, respectively). The final concentrations of glucose in mixed solutions were calculated as 2.91, 1.45, 0.73, 0.36, and 0.07 mg/mL, respectively. The absorbance of PANS with GOx and glucose was analyzed using the UV–Vis spectrophotometer.

## Results and Discussion

**Fig. 2:**
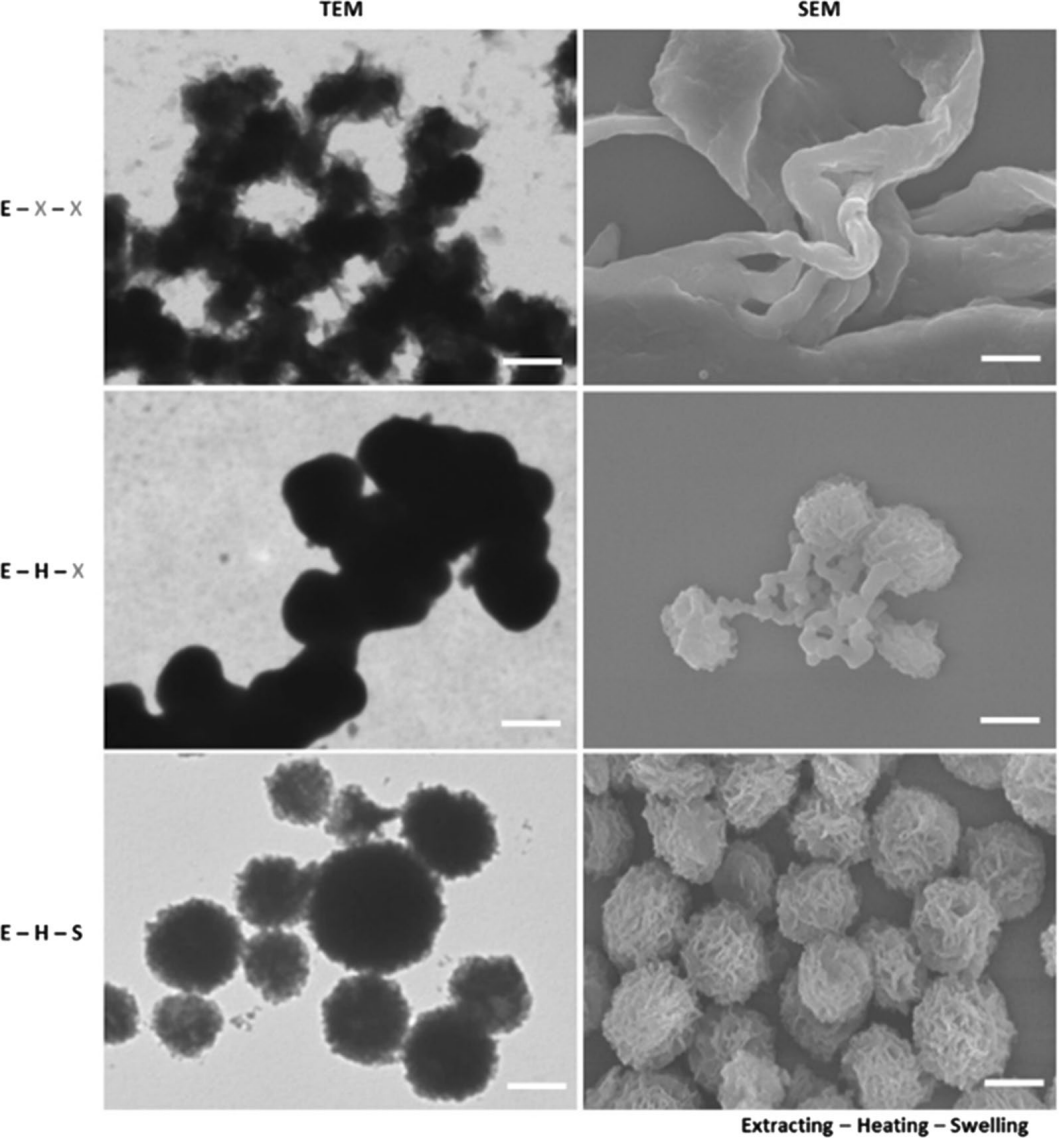
Morphology of PANS via transmission electron microscopic (TEM, left) and scanning electron microscopic (SEM, right) imaging. Alphabetic characters represent sequential synthetic processes of PANS; (E): extracting reactant with BE, (H): heating sequentially the reactant at 200 °C and 300 °C, and (S): swelling the reactant using EtOH. Note that (X): no treatment. Scale bars are 500 nm

The morphology of PANS was confirmed by transmission electron microscopic (TEM) and scanning electron microscopic (SEM) images as shown in Fig. 2. PANS exhibited a spherical shape with hierarchical structures. In the synthetic process of PANS, first, PAni was dissolved into NMP, and then, BE was added to the PAni/NMP solution. Because of the solubility difference for PAni between NMP and BE, PAni was extracted into the mixture, so this process was referred as the ‘extracting’ process. After the extracting process, PAni appeared fibrillar shape, and this shape may be generated due to intermolecular proximity between PAni molecules (the first row in Fig. 2 and see also Additional file 1: Fig. S1). After that, the mixture was heated sequentially at 200 °C for an hour and 300 °C for 30 min (‘heating’ process). The NMP has a boiling point at about 203 °C, so PAni existed in BE after the heating process. After the heating process, PAni was more nucleated than in the extracting process, and the shape of PANS became roundish. Moreover, the surface of PANS started to wrinkle, but many PANS particles were aggregated (the second row in Fig. 2 and see also Additional file 1: Fig. S1). Subsequently, PANS was washed as well as swelled using ethanol (EtOH) (the third row in Fig. 2 and see also Additional file 1: Fig. S1). After the swelling process, EtOH would be inserted between PAni molecules, so intermolecular distance also would be increased. Accordingly, PANS was existed at individual particles and also had a hierarchical structure. To confirm the ideal condition of formulation of PNAS, the concentration of PAni was also controlled (Additional file 1: Fig. S2). TEM image analysis according to feeding amount of PAni at each synthetic process (i.e., extracting, heating, and swelling process) was also conducted, and as feeding amount of PAni was decreased (from 50.0 mg/mL to 12.5 mg/mL), PANS was formulated much sparser. Moreover, the hierarchical structure of PANS was certainly confirmed via a 90°-tilted SEM image at feeding amount of PAni at 50 mg/mL (Additional file 1: Fig. S3). For the detailed studies of formulating mechanism of PANS, solubility test of PAni was preferentially conducted (Additional file 1: Fig. S4). PAni was known as hydrophobic and dissolved in a water-miscible polar aprotic solvent such as NMP [24]. The fibrillar shape of PAni was observed when PAni was dissolved in NMP. On the other hand, PAni in BE and EtOH had more aggregated and larger size than in NMP; furthermore, PAni in EtOH might be sparsely interacted with each PAni molecules. The solubility tests of PAni using measurements of absorbance spectra were also conducted; EtOH was shown the best solubility among the EtOH, BE, and deionized water (DW). The bare PAni molecules scarcely dissolved in DW. Moreover, another condition experiments were conducted according to molecular weight (Mw) of PAni (Additional file 1: Fig. S5). In the case of Mw 5 kDa, PANS exhibited more skein-like structures than other conditions (i.e., Mw 10 kDa and 50 kDa). In other cases, PAni did not nucleate such as Mw 5 kDa, and this phenomenon may be solubility differences to NMP for PAni. In another words, as the Mw of PAni is increased, PAni tends to insoluble in NMP, and thereby, PAni would not exist PAni nanofibers in NMP. As a result, sufficient PAni nanofibers were not existed, so skein-like structures of PANS also would not be formulated. From these results, the formulation of PANS is hypothesized as follows: (1) bare PAni does not dissolve in DW at all; (2) PAni has the best solubility in NMP; (3) PAni in NMP was extracted by the addition of BE via solubility difference; (4) the NMP was vaporized by heating process from PAni/(NMP and BE) mixture; (5) the resultant solution is washed by EtOH, and the EtOH is inserted between PAni molecules; (6) PANS particles are swelled and well-dispersed in DW.

**Fig. 3:**
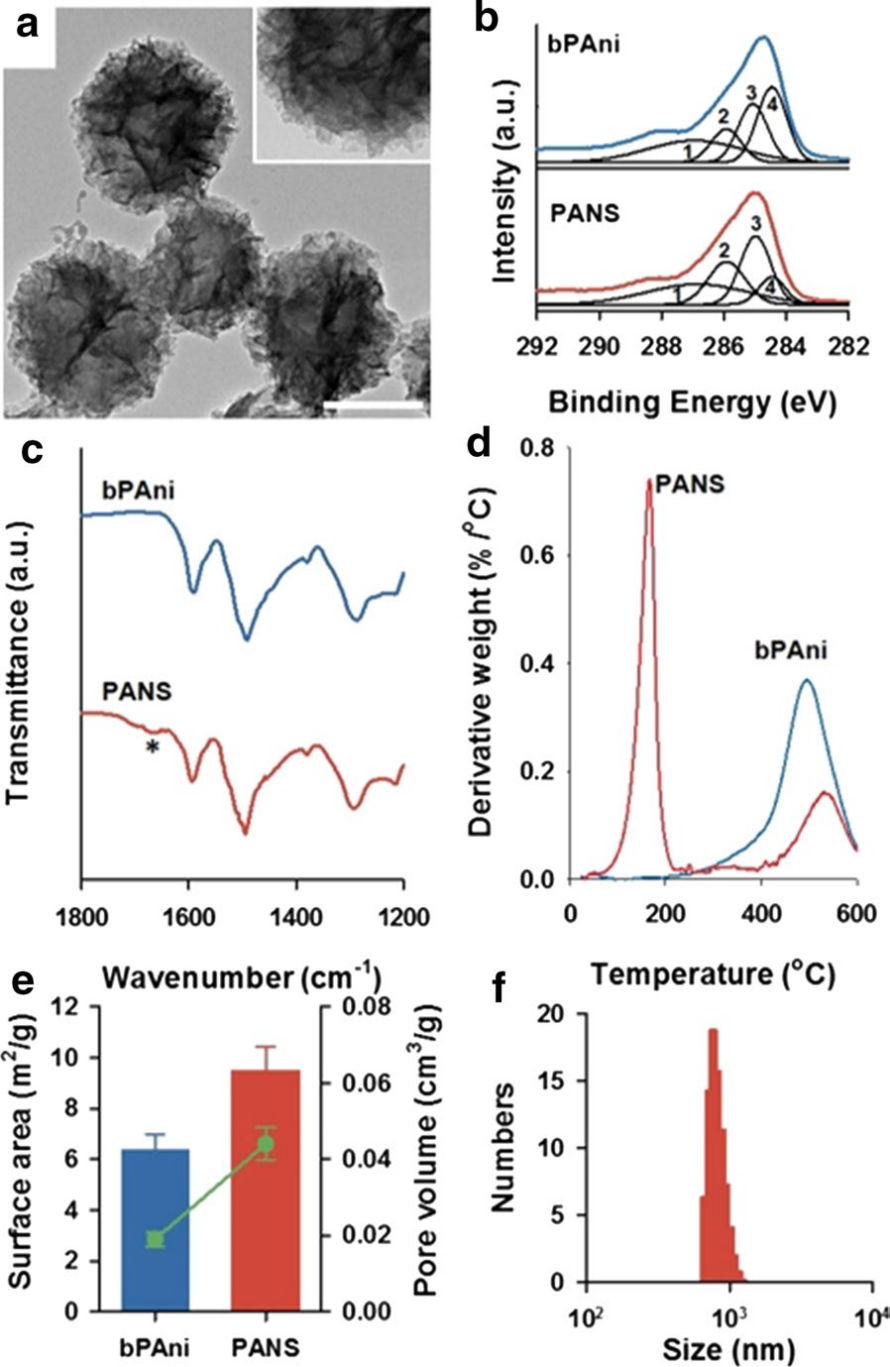
**a** High-resolution transmission electron microscopic (HRTEM) images of PANS. Scale bar is 100 nm, and inset is a high magnification image. **b** XPS C1s spectra of bare polyaniline (bPAni) (upper) and PANS (lower). 1: C=O/C–O, 2: C–N^+^/C=N^+^, 3: C–N/C = N, 4: C–C/C–H. **c** FTIR spectra of bPAni (upper) and PANS (lower). An asterisk represents a interesting peak described in more detail in the text. **d** Thermogravimetric analysis (TGA) of bPAni (upper) and PANS (lower). **e** Surface area (bar) and pore volume (line and scatter) of bPAni and PANS using Brunauer–Emmett–Teller (BET) analysis. **f** Size distribution of PANS via dynamic light scattering (DLS) method

To study the characteristics of PANS, first of all, high-resolution TEM (HRTEM) imaging analysis was conducted (Fig. 3a). PANS had morphology for a ball of yarn, which was composed of many PAni nanofibers. To verify the molecular structure of PANS, X-ray photoelectron spectroscopic (XPS) analysis for bare PAni (bPAni) and PANS was conducted (Fig. 3b and see also Additional file 1: Fig. S6). The C1s core level spectra can be deconvoluted in four peaks: C=O/C–O at 286.7 eV (1), C=N + /C–Cl at 285.8 eV (2), C-N/C=N at 285.0 eV (3), and C–C/C–H at 284.4 eV (4), respectively. Deconvolution of C1s was based on data reported earlier by Golczak et al. [30]. In particular, the changes of distributions for (3) and (4) were remarkable, and these result mean that nitrogen portion was increased in PANS. This phenomenon may be attributed to the existence of NMP in PANS structures, and this NMP molecule affects interactions between PAni molecules for the formulation of PANS. To further investigate for molecular structure of PANS, Fourier transform infrared (FTIR) spectra for bPAni and PANS were also analyzed (Fig. 3c and see also Additional file 1: Fig. S7). The peaks at 1290 cm^−1^ (aromatic C–N stretching), 1490 cm^−1^ (C=C and C=N stretching of benzenoid ring), and 1590 cm^−1^ (C=C and C=N stretching of quinoid ring) were confirmed in both of cases for bPAni and PANS. The specific peaks of PANS at 1670 cm^−1^ (asterisk in Fig. 3c) might be attributed to tertiary amine in NMP, and this peak was also observed in FTIR spectrum of NMP (Additional file 1: Fig. S7). Thermogravimetric analysis (TGA) results of bPAni and PANS reveal that PANS was composed of not only PAni but also other molecules (Fig. 3d). The degradation of bPAni was observed at about 350 °C; however, the degradation point of PANS was observed at about 200 °C, which coincides with the boiling point of NMP. From XPS to TGA results, Fig. 3b–d reveals that PANS was not only composed of pure PAni, but also consisted of PAni and other organic compounds such as NMP. Additionally, surface area and pore volume of PANS were determined by Brunauer–Emmett–Teller (BET) analysis (Fig. 3e), and PANS had 9.486 ± 0.728 m^2^/g of surface area and 0.044 ± 0.004 cm^3^/g of pore volume. (c.f. 6.358 ± 0.682 m^2^/g of surface area and 0.019 ± 0.001 cm^3^/g of pore volume for bPAni, respectively.) These results mean that PANS is more porous with high surface-to-volume ratio than bare PAni. The distribution of hydrodynamic diameter of PANS exhibited 799 ± 85 nm in an aqueous solution (Fig. 3f). These results in Fig. 3 show that PANS is solely composed of organic compounds (PAni and NMP) with hierarchical structures.

**Fig. 4:**
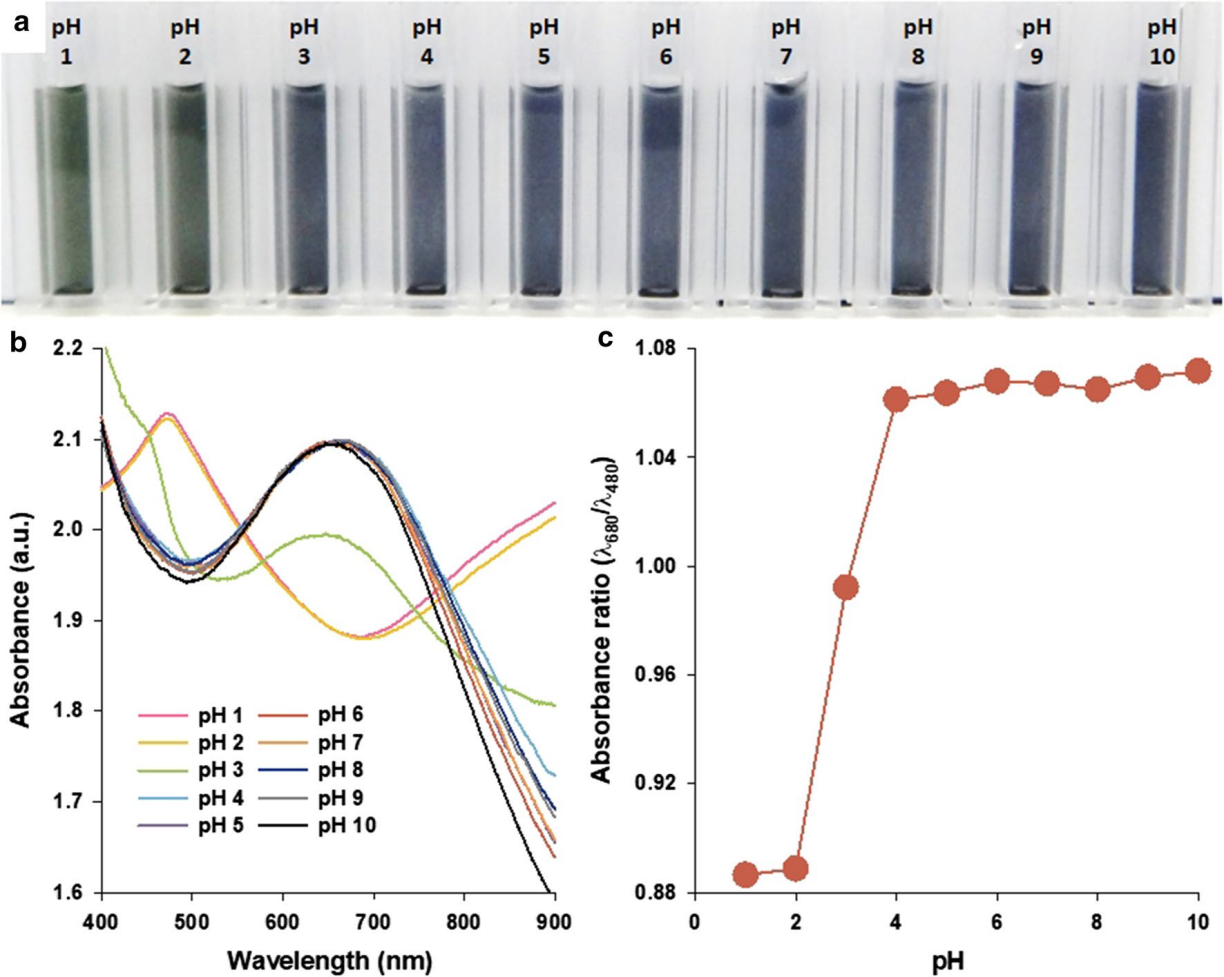
**a** A photograph, **b** absorbance spectra, and **c** absorbance ratio for representative wavelengths for EB (at 680 nm) and ES (at 480 nm) states of PANS solutions in indicated pH conditions

To investigate the possibility of PANS as convertible optical probes, color changes of PANS were analyzed using absorbance spectra. The changes of absorbance properties of PAni structures, such as nanoparticles, films, and sheets are well known as varying its doping/dedoping states. The doping/dedoping states of PAni can be adjusted by various dopants, such as strong acids, Lewis acids, transitional metals, and alkali ions [31, 32]. PANS also exhibited those properties according to changes of surrounding pH values (Fig. 4). At low pH levels (< pH 2), PANS transitioned to an emeraldine salt (ES, green color) state, as indicated by the presence of the π–π* transition of the benzenoid rings as well as polaron band transitions at about 480 and 800–900 nm, respectively [33]. With increasing pH values, the peaks at 480 and 800–900 nm are gradually decreased in absorbance, and a strong absorbance peak at about 680 nm is observed. The absorbance peak at 680 nm is attributed to excitation from the highest occupied molecular orbital of the three-ring benzenoid part of the PAni to the lowest unoccupied molecular orbital of the localized quinoid ring and the two surrounding imine nitrogens in the emeraldine base (EB) state of the PAni [34]. To more accurately distinguish between ES and EB state of PANS, the absorbance ratio (*λ*_680_/*λ*_480_), was calculated at representative wavelength positions of peaks at EB and ES states of PANS, respectively (Fig. 4c). As the pH value decreased from 10 to 4, the absorbance ratio maintained at nearly 1.06, and it decreased dramatically to nearly 0.88 at pH values from 4 to 1. These results indicate that spectral changes of PANS can be used to evaluate the specific changes of redox state by changes of surrounding environments.

**Fig. 5:**
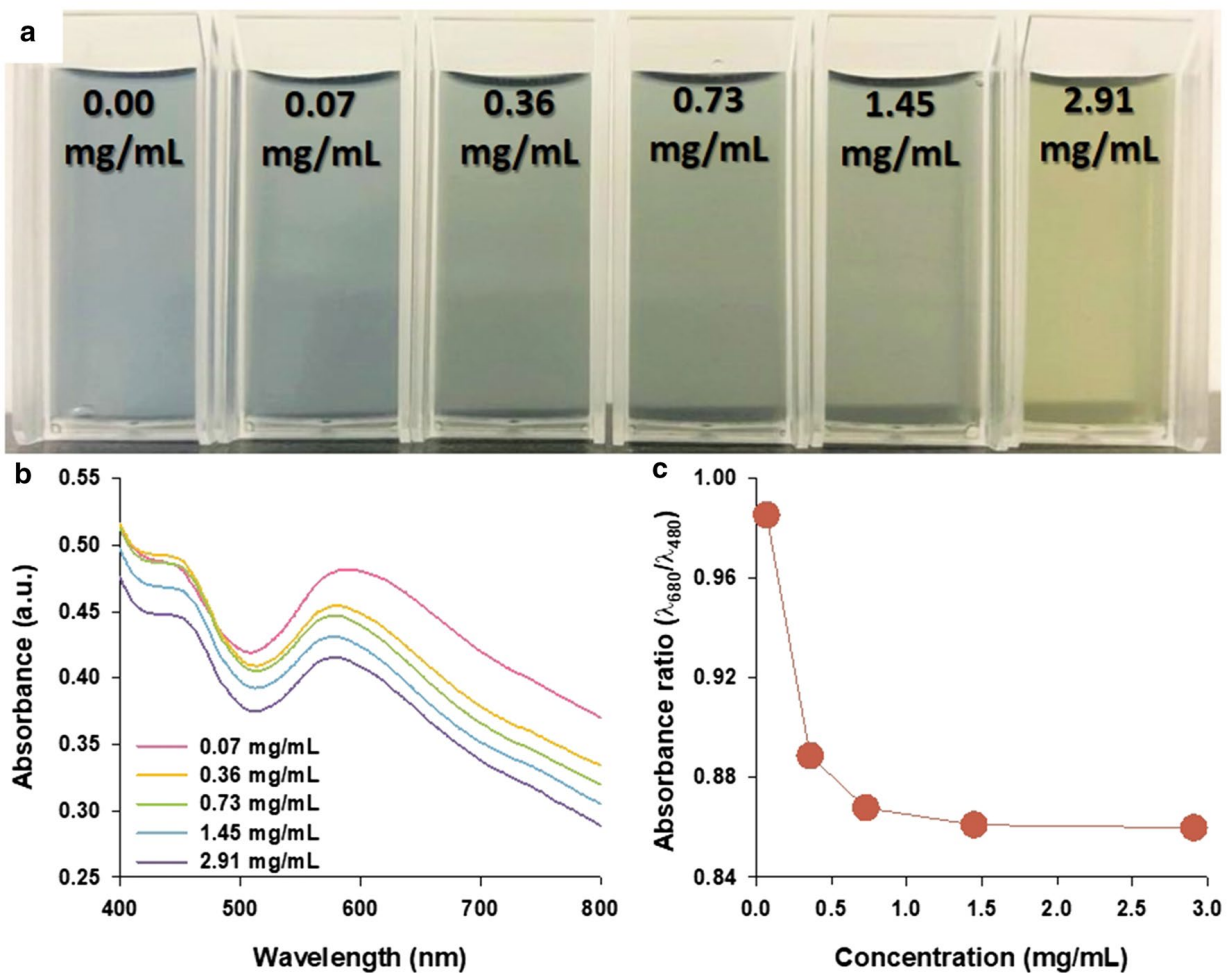
**a** A photograph, **b** absorbance spectra, and **c** absorbance ratio for representative wavelengths for EB (at 680 nm) and ES (at 480 nm) states of PANS solutions with indicated glucose concentrations

To investigate the sensing capability of PANS for specific changes of redox state from the changes of surrounding environments, glucose and GOx were selected as representative candidates. As increasing the concentrations of glucose from 0.00 to 2.91 mg/mL, the color of PANS solutions changed from blue to green (Fig. 5a). The absorbance spectra of PANS were also analyzed (Fig. 5b), but shape changes in spectra were less than those in experiments with pH changes as shown in Fig. 4. This phenomenon can be explained by the following equations:

1 $${\text{Glucose}} + {\text{H}}_{2} {\text{O}} + {\text{O}}_{2} \mathop{\longrightarrow}\limits^{{\text{GOx}}}{\text{Gluconic}}\,{\text{acid}} + {\text{H}}_{2} {\text{O}}_{2}$$

2 $${\text{H}}_{2} {\text{O}}_{2} + {\text{PANS}}\,({\text{EB}}) \to 2{\text{e}}^{ - } + {\text{O}}_{2} + {\text{PANS}}\,({\text{ES}})$$

In Eq. (1), glucose is oxidized by GOx, resulting in gluconic acid and hydrogen peroxide. Moreover, as shown in Eq. (2), the resulting hydrogen peroxide is reduced the PANS in EB state to ES state. However, the values of absorbance ratio (*λ*_680_/*λ*_480_) were formed between 0.98 and 0.86, and this result indicates that PANS is existed at intermediated state (*λ*_680_/*λ*_480_ = 0.98) and ES state (*λ*_680_/*λ*_480_ = 0.86) as shown in Fig. 5c. The reaction between glucose and GOx is representative redox reaction, and this reaction is well known for producing hydrogen peroxide, resulting in hydrogen ions and electrons. These results indicate that PANS can be doped with hydrogen ions and electrons. As a result, the π–π* transition of the benzenoid ring is observed in absorbance spectra through the formation of shoulder at 480 nm. Collectively, this result indicates that PANS can be used to detect enzymes such as glucose. It is thought that PANS could also be used to detect the activity of other enzymes and to confirm redox activity within biological system.

## Conclusion

In summary, the formulation of skein-shaped PANS, which is composed of PAni fibers, has been achieved, and the formulating mechanism has been also proposed. The synthetic process of PANS contains sequential extracting, heating, and swelling processes. The compositions of PANS have been analyzed, which composed of solely organic materials. Moreover, the possibilities of PANS as convertible optical probes for sensing of surrounding redox states changes were confirmed via the change of pH values and combination of glucose and GOx. The present study sheds light on the synthesis of new class of 3D shaped nanostructures as well as nanobiosensors for sensing of surrounding redox state changes.

## Supplementary information

**Additional file 1**. Addtional experiments of control group and characterization results.

## Data Availability

All data generated or analyzed during this study are included in this published article.
