# Supplementary material for: Polyaniline Nanoskein: Synthetic Method, Characterization, and Redox Sensing
Source: Nanoscale Res Lett. 2020 Nov 13;15:215. doi: 10.1186/s11671-020-03446-2 (PMC7666266; doi:10.1186/s11671-020-03446-2)
Supplement: Supplementary file 1 — Additional file 1. Addtional experiments of control group and characterization results. [file 11671_2020_3446_MOESM1_ESM.docx]

**Supporting Information**

**Polyaniline Nanoskein: Synthetic Method, Characterization, and Redox Sensing**

Yoochan Hong^a^, Hyun Soo Kim^a^, Taeha Lee^b^, Gyudo Lee^b^, and Ohwon Kwon^a*^

^a^Department of Medical Devices, Korea Institute of Machinery and Materials (KIMM), Daegu 42994, Republic of Korea

^b^Department of Biotechnology and Bioinformatics, Korea University, Sejong 30019, Republic of Korea

**^*^Corresponding Author:**

Dr. Ohwon Kwon

Department of Medical Devices, Korea Institute of Machinery and Materials (KIMM), Daegu 42994, Republic of Korea

*Tel:* 82-53-670-9001; *Fax:* 82-53-670-9053; *E-mail address:* owkwon@kimm.re.kr


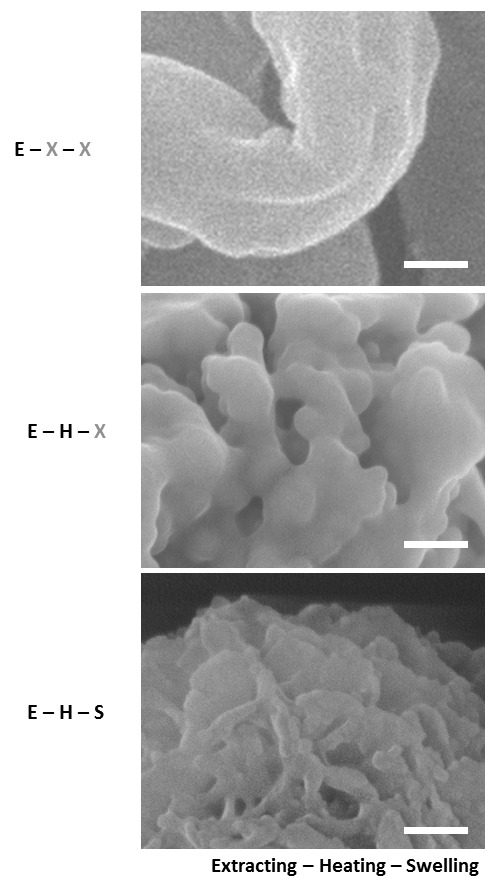


**Fig. S1.** FESEM images of PANS. Alphabetical characters represent sequential synthetic processes of PANS; (E): extracting reactant with BE, (H): heating sequentially the reactant at 200°C and 300°C, and (S): swelling the reactant using EtOH. Note that (X): no treatment. Scale bars are 100 nm.


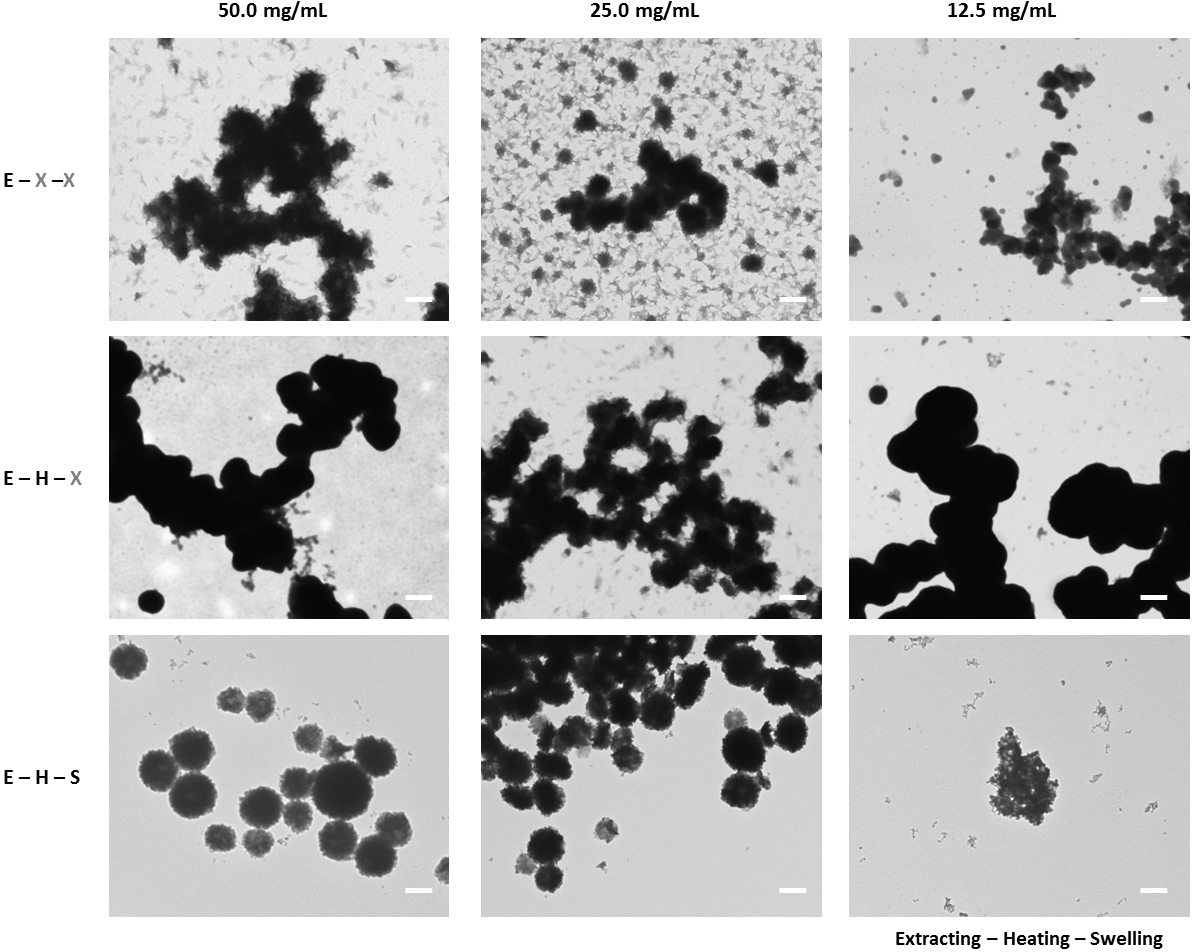


**Fig. S2.** TEM images of PANS according to changes of feeding amounts of PAni. Alphabetical characters represent sequential synthetic processes of PANS; (E): extracting reactant with BE, (H): heating sequentially the reactant at 200°C and 300°C, and (S): swelling the reactant using EtOH. Scale bars are 500 nm.


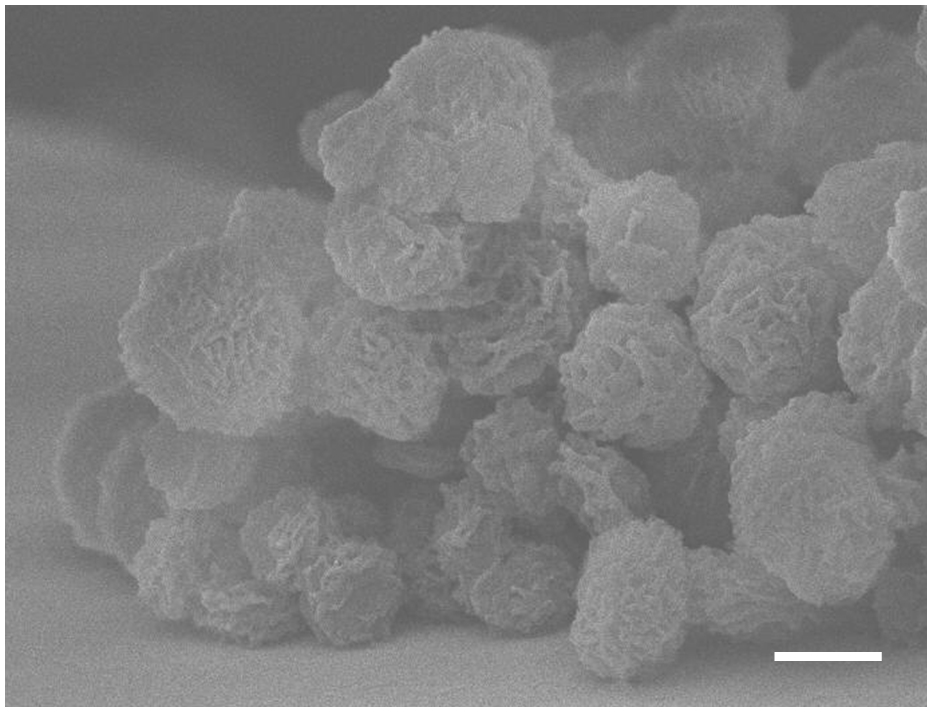


**Fig. S3.** A 90°-tilted FESEM image of PANS. Scale bar is 500 nm.


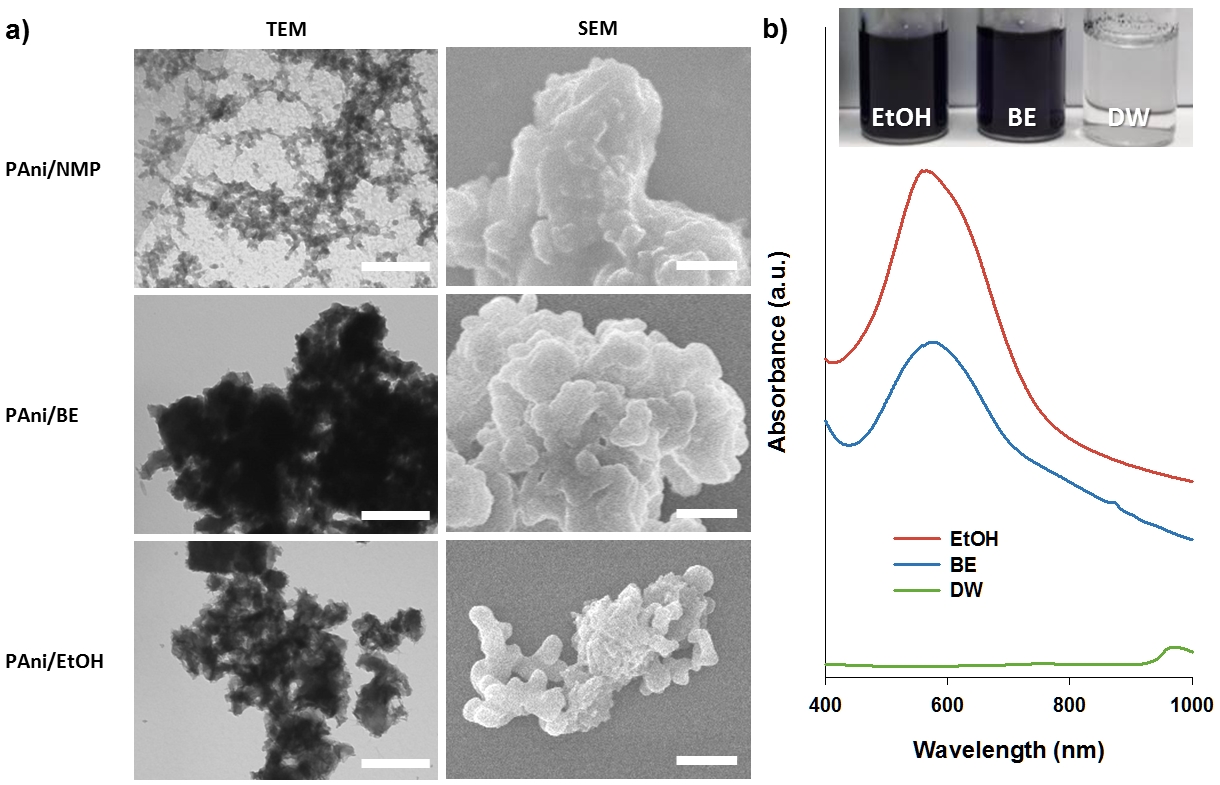


**Fig. S4.** a) TEM and SEM images of PAni just dispersed in indicated solvents. Scale bars are 500 nm. b) Absorbance spectra of PAni dispersed in indicated solvents. Inset is a photograph of PAni solutions dispersed in indicated solvents.


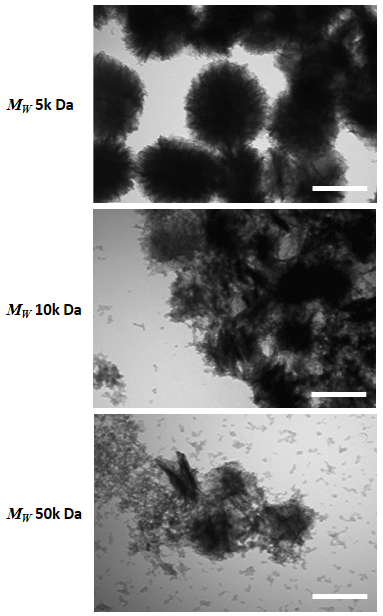


**Fig. S5.** TEM images of PANS according to changes of molecular weight (*M_W_*) of PAni. Scale bars are 200 nm.


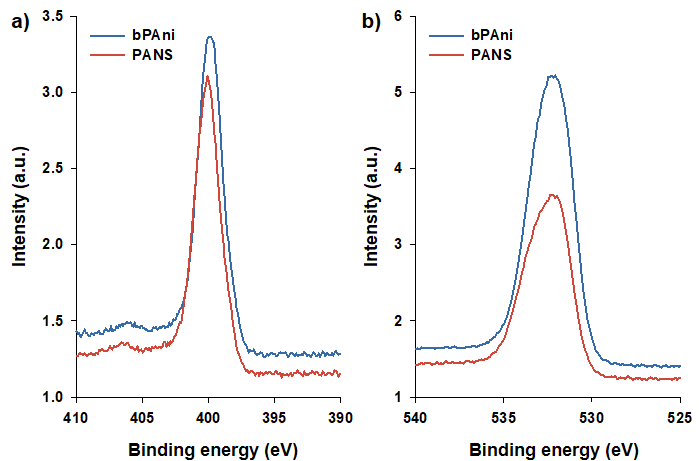


**Fig. S6.** XPS spectra of a) N_1s_ and b) O_1s_ for bPAni and PANS, respectively.


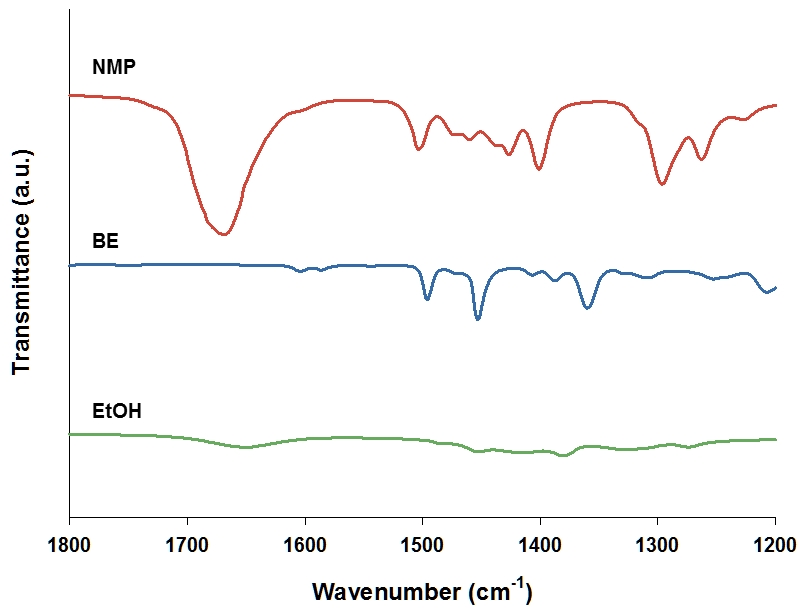


**Fig. S7.** FTIR spectra of NMP, BE, and EtOH.
